# Genome mining unveils widespread natural product biosynthetic capacity in human oral microbe *Streptococcus mutans*

**DOI:** 10.1038/srep37479

**Published:** 2016-11-21

**Authors:** Liwei Liu, Tingting Hao, Zhoujie Xie, Geoff P. Horsman, Yihua Chen

**Affiliations:** 1 State Key Laboratory of Microbial Resources, Institute of Microbiology, Chinese Academy of Sciences, Beijing, 100101, China; 2 University of Chinese Academy of Sciences, Beijing, 100049, China; 3 Department of Chemistry and Biochemistry, Wilfrid Laurier University, Waterloo, ON, N2L3C5, Canada

## Abstract

*Streptococcus mutans* is a major pathogen causing human dental caries. As a Gram-positive bacterium with a small genome (about 2 Mb) it is considered a poor source of natural products. Due to a recent explosion in genomic data available for *S. mutans* strains, we were motivated to explore the natural product production potential of this organism. Bioinformatic characterization of 169 publically available genomes of *S. mutans* from human dental caries revealed a surprisingly rich source of natural product biosynthetic gene clusters. Anti-SMASH analysis identified one nonribosomal peptide synthetase (NRPS) gene cluster, seven polyketide synthase (PKS) gene clusters and 136 hybrid PKS/NRPS gene clusters. In addition, 211 ribosomally synthesized and post-translationally modified peptides (RiPPs) clusters and 615 bacteriocin precursors were identified by a combined analysis using BAGEL and anti-SMASH. *S. mutans* harbors a rich and diverse natural product genetic capacity, which underscores the importance of probing the human microbiome and revisiting species that have traditionally been overlooked as “poor” sources of natural products.

Over 60% of drugs on the market are derived from natural products, and 38% are of microbial origin, primarily from actinobacteria and fungi^1^,^2^. The last three decades have witnessed tremendous progress in elucidating the biosynthetic mechanisms of varied microbial natural products including compounds synthesized by polyketide synthases (PKSs), nonribosomal peptide synthetases (NRPSs), and the ribosomally synthesized and post-translationally modified peptides (RiPPs). This genes-to-molecules foundational knowledge has enabled the identification of cryptic natural product gene clusters from microbial genomes and facilitated genomics guided discovery of novel natural products.

PKSs are usually classified into three types: type I PKSs are large modular proteins containing various functional domains; type II PKSs are single or bifunctional proteins used in an iterative fashion; and type III PKSs employ coenzyme A-tethered substrates instead of the acyl carrier proteins (ACP) recruited by types I and II PKS systems. A typical type I PKS module minimally contains acyltransferase (AT), ACP (also called thiolation domain, T) and ketosynthase (KS) domains. Additional PKS domains include ketoreductase (KR), dehydratase (DH), enol reductase (ER) as well as some rare functional domains that together generate an extraordinary diversity of polyketide skeletons^3^,^4^. Similar to type I PKSs, NRPSs are also modular proteins minimally comprising adenylation (A), peptidyl carrier protein (PCP, also called thiolation domain T) and condensation (C) domains. Examples of additional functional domains include cyclase (Cy), epimerase (E) and methyltransferase (MT) domains^5^. A thioesterase (TE) domain or a terminal reductase (R) domain releasing the mature polyketide or polypeptide chain from the biosynthetic assembly line is commonly found at the carboxyl terminus^6^. In addition, a phosphopantetheinyl transferase (PPTase) is necessary to type I and II PKS and NRPS proteins, which converts them from apo- to functional holo-form by transferring a phosphopantetheinyl arm from coenzyme A to the Ser residue of their thiolation domains^7^.

RiPPs represent a large family of natural products derived from ribosomally synthesized polypeptide precursors, which usually consist of an N-terminal leader peptide and a core peptide. The leader peptide is essential for guiding post-translational modification enzymes and transporters acting on the precursor peptide, and the core peptide becomes the final product after removal of the leader peptide by proteases^8^. The bacteriocins are an important group of RiPPs that can be classified as either modified or unmodified based on post-translational modifications to their core peptides. To date, a number of bacteriocins with diverse structures and bioactivities are generated by the action of various modification enzymes such as Ser/Thr dehydratases, methyltransferases, and macrocylases^9^,^10^,^11^,^12^.

As microbial genome sequence space rapidly expands, a genomics guided approach is increasingly important for natural product discovery. Analysis of diverse bacterial genomes demonstrated statistically that the number of natural product gene clusters in a strain increases with the size of its genome, implying that bacteria with genome sizes less than 2 Mb rarely contain polyketide and nonribosomal peptide biosynthetic gene clusters and are poor sources of natural products^13^,^14^. Consequently, natural product genome mining efforts have focused almost exclusively on bacteria with large genomes such as cyanobacteria and actinomycetes from terrestrial or aquatic environments^15^,^16^,^17^. Until recently the human microbiome has seldom been considered as a source of natural products. However, in silico analysis of thousands of bacterial genomes from the human microbiome revealed that the gut and oral cavity are the two richest habitats for bacteria harboring numerous natural product gene clusters. These microbes are distributed across diverse bacterial families, including some with small genome sizes, which suggests that some human-associated bacteria with small genome sizes could represent an overlooked source of natural products^18^.

*Streptococcus mutans* is a Gram-positive facultative anaerobe considered as a major contributor to dental cavities in humans and animals. Acid resistance, biofilm formation, and production of bacteriocins are some of the characteristics that contribute to successful colonization and proliferation of *S. mutans* in the human oral cavity. Several bioactive compounds have recently been isolated from *S. mutans*, including mutanobactins and mutacins I-IV^19^,^20^,^21^,^22^,^23^,^24^. Mutanobactins are compounds of hybrid PKS/NRPS origin that inhibit the morphological transition of *Candida albicans*, an opportunistic human pathogen^19^. Mutacins I-IV are bacteriocins exhibiting antibacterial activity against closely related species and some other Gram-positive bacteria^25^.

Despite the isolation of several compounds from *S. mutans*, this species has seldom been considered as a potential source of natural products due to its relatively small genome size (about 2 Mb). Recent genome sequencing projects have provided a great deal of genomic data for many strains of *S. mutans* that enables a comprehensive survey of its potential natural product biosynthetic capacity. Herein we report the identification of 355 natural product gene clusters from 169 *S. mutans* genomes, which reveal this species as a rich source of natural products in the human microbiome and serves as an important reminder to re-examine the natural product biosynthetic capacity of bacteria that have been previously neglected due to small genome sizes.

## Results

### An overview of natural product biosynthetic gene clusters in *S. mutans*

A total of 169 *S. mutans* genomes were analyzed with anti-SMASH and BAGEL, which identified 355 natural product gene clusters distributed among 138 *S. mutans* strains (Supplementary Tables SI, SII and SIII). The identified natural product gene clusters belonged to NRPS (1), PKS (7), PKS/NRPS hybrid (136) and RiPP (211) families (Supplementary Tables SI and SII). The number of gene clusters in each positive *S. mutans* genome varied from one to five, with 66 (39%) strains containing more than two (Supplementary Table SI).

Natural product gene clusters possessing modular proteins (including NRPS, PKS and hybrid PKS/NRPS) were found in 114 (67.4%) strains, 29 (17.2%) of which harbored no less than two gene clusters. For example, the six strains *S. mutans* 4SM1, *S. mutans* 4VF1, *S. mut*ans 15VF2, *S. mutans* 1SM1, *S. mutans* NLML1 and *S. mutans* SA38 contained one PKS gene cluster and one hybrid PKS/NRPS gene cluster, and three clusters (1 PKS, 1 NRPS and 1 hybrid PKS/NRPS) were discovered in *S. mutans* B24Sm2 occupying 5.4% of its 2.28 Mb genome.

In addition, 211 RiPPs gene clusters were also detected, including 32 modified bacteriocin (lantibiotic) gene clusters from 28 strains and 179 unmodified bacteriocin gene clusters from 116 strains (Supplementary Table SII). All of the modified bacteriocin gene clusters were typical lantibiotic gene clusters, and three strains, *S. mutans* R221, *S. mutans* ST1 and *S. mutans* B107SM-B had more than one lantibiotic gene clusters. At least two unmodified bacteriocin gene clusters were identified in 46 (27.2%) strains.

### The hybrid PKS/NRPS gene clusters in *S. mutans*

**Figure 1:**
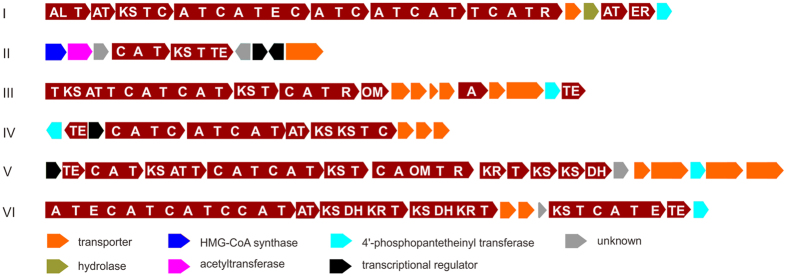
The 136 hybrid PKS/NRPS gene clusters identified in this study. The functional domains in NRPS and PKS proteins are indicated in bold: A, adenylation domain; AL, loading domain; AT, acyl-transferase; C, condensation domain; DH, dehydratase; E, epimerization; KR, ketoreductase; KS, ketosynthase; OM, *O*-methyltransferase; R, terminal reductase; T, thiolation domain (including both peptidyl carrier protein and acyl carrier protein); TE, thioesterase. The other functional enzymes and transporter proteins are indicated by arrows with different colors.

Based on gene composition and organization, 136 identified hybrid gene clusters ranging from 12.6 kb to 36.2 kb in length were classified into six groups shown in Fig. 1, Supplementary Tables SI and SIII. Group I consists of 58 homologous hybrid PKS/NRPS gene clusters sharing greater than 78% nucleotide sequence identity. The gene clusters in this group contain 12 genes including one loading domain, one PKS module, six NRPS modules and a set of tailoring enzymes at the end of this cluster. The only known products of group I are the aforementioned mutanobactins isolated from *S. mutans* UA159^19^,^20^,^26^, implying that similar compounds are encoded by group I gene clusters.

**Figure 2:**
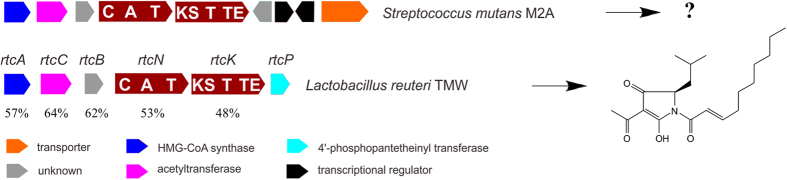
The biosynthetic gene cluster of reutericyclin in *Lacterobacillus reuteri* TMW and one example of the group II hybrid PKS/NRPS gene clusters from *S. mutans* M2A. The amino acid identities between two gene clusters are shown under the genes of the reutericyclin cluster. The functional domains in PKSs and NRPSs are indicated in bold: A, adenylation domain; C, condensation domain; KS, ketosynthase; T, thiolation domain; TE, thioesterase. The other functional enzymes and transporters are indicated by arrows with different colors.

In total, 22 gene clusters belonging to group II were identified, and these possessed five genes encoding proteins with more than 50% identity to homologs in the reutericyclin gene cluster from *Lactobacillus reuteri* TMW1.106, TMW1.112 and TMW1.656 (Fig. 2). Genes *rtcN* and *rtcK* were proposed to respectively encode an NRPS and a PKS that catalyze installation of the core structure of reutericyclin; genes *rtcABC* encode an acyltransferase cassette responsible for the addition of an acetyl group^27^. The high similarity of sequence and gene organization suggests that the group II gene clusters encode a compound similar to reutericyclin (Fig. 2). Furthermore, four extra genes in group II clusters encode two regulatory proteins, one transporter, and one protein of unknown function that might be involved in regulation, resistance or modification of the end product.

The third group consists of 29 hybrid PKS/NRPS gene clusters. Three modular proteins encoded by group III clusters comprise two PKS modules, three NRPS modules and a terminal reductase domain for a reductive release of the thioester-tethered product. Group III clusters also encode six transporters, an O-methyltransferase, an adenylation domain, a PPTase and a type II TE that may function in an editing role to hydrolyze improperly loaded elongation units from the thiolation domains.

Groups IV and V hybrid PKS/NRPS gene clusters are always present concomitantly and were identified in four strains: *S. mutans* NMT4863, *S. mutans* N29, *S. mutans* 5SM3 and *S. mutans* B112SM-A. Group VI hybrid PKS/NRPS gene clusters have 19 members, and each is composed of four genes encoding modular proteins with totally five NRPS modules and three PKS modules. Genes encoding AT, TE, transporter and PPTase proteins are also found in the group VI clusters. Significantly, hybrid PKS/NRPS gene cluster groups III-VI are unlike any known clusters with identified products, indicating that they produce novel compounds that await characterization.

### The NRPS gene clusters in *S. mutans*

**Figure 3:**
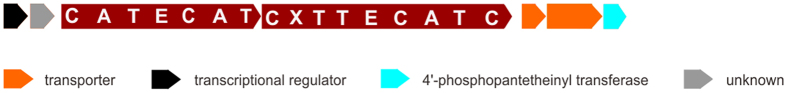
*S. mutans* B24Sm2 possesses one NRPS gene clusters identified by anti-SMASH. NRPS functional are indicated in bold: A, adenylation domain; C, condensation domain; E, epimerase domain; T, thiolation domain; X, HXXPF repeat domain. The other functional enzymes and transporter proteins are indicated by arrows with different colors.

Only one NRPS gene clusters were identified from 169 *S. mutans* genomes, and it was discovered in *S. mutans* B24Sm2 (Fig. 3). This new NRPS gene cluster comprises seven genes, two of which encode large NRPS complexes possessing four modules. Specifically, the third NRPS module is missing of the adenylation domain, but has a functionally unknown HxxPF repeat domain in front of two repeated T domains. A condensation domain is found at the C-terminus instead of a TE or a reductase domain, suggesting that the peptide product may be released from the NRPS machinery by a condensation reaction as reported previously^28^.

### The PKS gene clusters in *S. mutans*

**Figure 4:**
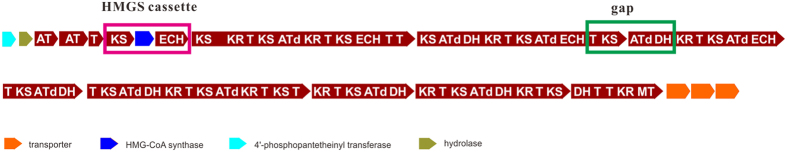
Detection by anti-SMASH of seven PKS gene clusters from 169 *S. mutans* genomes. Two complete gene clusters were found from *S. mutans* A38 and B24Sm2. The other five gene clusters, which are cut off by gaps marked with a green rectangle, were found in *S. mutans* 4VF1, 4SM1, 15VF2, 1SM1 and NLML1. The HMGS cassette found in the PKS gene clusters is enclosed by a pink rectangle. The functional domains in PKS proteins are indicated in bold: AL, loading domain; AT, acyltransferase; ATd, AT docking site; DH, dehydratase; ECH, enoyl-CoA hydratase; MT, methyltransferase; KR, ketoreductase; KS, ketosynthase; T, thiolation domain. The other functional enzymes and transporters are indicated by arrows with different colors.

Only one group of type I PKS gene cluster was identified, which distributes in 7 of the 169 analyzed strains and has two versions (Fig. 4). In *S. mutans* A38 and *S. mutans* B24Sm2, the PKS gene clusters span about 70 kb and contain 8 genes encoding proteins with 14 AT-less (or trans-AT) PKS modules. In AT-less PKS systems, the PKS modular proteins possess AT docking domains (ATd) that serve as locations for interaction with the free-standing AT that loads buliding blocks to the thiolation domain^29^. Surprisingly, a methyltransferase domain replaces the typical TE or R domain at the C-terminus of the PKS, indicating an unusual polyketide chain release mechanism. In addition to the modular PKS genes, a PPTase gene was found at the 5′-end followed by a putative hydrolase and HMG-CoA synthase cassette for installing branched polyketide chains^30^. Three transporters encoded by genes at the 3′-end of the cluster may export the product out of the cell. The other five strains (*S. mutans* 4VF1, *S. mutans* 4SM1, *S. mutans* 15VF2, *S. mutans* NLML-1 and *S. mutans* 1SM1) harbor PKS clusters that are split into two parts by a gap (green box in Fig. 4) that normally contains the ACP (T) and KS domains of the second modular protein and the ATd and DH domains of the third modular protein. Because only draft sequence data are available for these five strains and the gaps are all at the ends of the sequencing contigs, it is most likely that the five PKS clusters are split artificially.

### Streptococcal distribution of NRPS and PKS genes

A careful analysis of the A-T-C/KS-AT-T module existing in NRPS, PKS, and hybrid PKS/NRPS gene clusters in the genomes of 10,038 *Streptococcus* strains from the Entrez Protein database, which is limited to genome-annotated *Streptococcus spp.* from the NCBI database (up to April 18, 2016), showed that about 12.1% of the A-T-C modules and 10.5% KS-AT-T module are found in 169 *S. mutans* genomes (Supplementary Table SIV). The result revealed an uneven distribution of NRPS, PKS and hybrid PKS/NRPS clusters in different genera of *Streptococcus*; *S. mutans* is about 7-fold enriched in A-T-C modules and 6-fold enriched KS-AT-T modules compared to the *Streptococcus* average. This implies that *S. mutans* may be an exceptional natural product producer among a plethora of relatively barren *Streptococci*.

### The lantibiotic gene clusters in *S. mutans*

**Figure 5:**
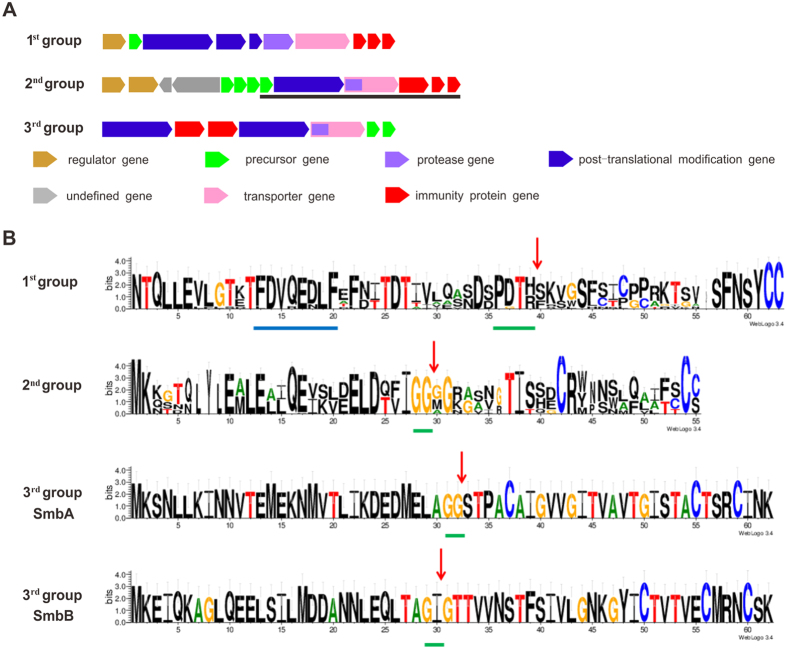
Composition of the lantibiotic gene clusters in *S. mutans* (**A**) and alignment of the lantibiotic precursors (**B**). (**A**) The putative lantibiotic gene clusters classified into three groups based on thioether-forming genes, *LanBC* and *LanM*. The genes are indicated by arrows with different colors. (**B**) The conserved motifs in class I lantibiotic leader peptides are underlined in blue. The green underline indicates the conserved sites in front of the cleavage sites, which are marked by red arrows. This figure was generated by web-based software WebLogo 3^31^.

We identified 32 gene clusters encoding modified RiPPs that all belonged to the lantibiotic family and could be classified into three groups based on the modification genes *LanBC* and *LanM*, which catalyze thioether ring formation (Fig. 5 and Supplementary Table SII)^31^. The first group contains the *lanBC* genes and an independent protease, in contrast to the transporter protease domain found in the other two groups. The five modified lantibiotic gene clusters belonging to the first group are highly conserved in both gene composition and organization, with the exception that *S. mutans* B88SM-A and *S. mutans* 66–2 A have only one precursor gene instead of two. The first group gene clusters are very similar to those encoding mutacins I and III from *S. mutans* CH43 and UA789, but possess more different precursor peptides (Fig. 5 and Supplementary Figure SI)^21^,^22^. Aligning 18 of the group I precursor peptides with Weblogo 3.0 revealed that the characteristic motif “F(N/D)LD” of class I lantibiotic leader peptides exists as a variant “FDVQ” motif here^32^. Notably, it was proposed that the “FDVQ” motif of the mutacin 1140 (S3) leader peptide is not required for its maturation, but a new “EDLF” box following “FDVQ” was important for recognition by post translational modification enzymes^33^. Interestingly, both the “FDVQ” and “EDLF” motifs and the cleavage site after the “PDT(R/H)” box, which was characterized in mutacin 1140, are well conserved in all leader peptides of the first group lantibiotic precursors, indicating a conserved and potentially unique post translational modification.

The second group of lantibiotics, possessing one *lanM* for thioether formation instead of *lanBC*, was identified in 20 gene clusters. Among them, five complete gene clusters containing all 13 genes were identified, while the other 15 clusters are comprised of only the 6 genes at the 3′ end of the cluster (Fig. 5, underlined in black). Once again, we cannot rule out truncation due to poor quality of the draft genome sequences, since all the truncated clusters are at the ends of the contigs. The gene organization of the complete clusters in the second group are very similar to the mutacin K8 gene cluster (Supplementary Figure SI) with the exception of the *S. mutans* B082SM-A cluster possessing only two precursor genes instead of four^34^. An alignment of all 34 precursor peptides revealed a conserved “GG” cleavage site that is characteristic of this group (Fig. 5).

The third group consists of 7 clusters that encode modified RiPPs and possess two putative *lamM* genes. The cluster from *S. mutans* GS-5 is known to produce mutacin Smb by the action of two components, SmbA and SmbB^35^. The remaining six pairs of precursors are identical to SmbA and SmbB, revealing strong conservation among this group of modified bacteriocins.

**Figure 6:**
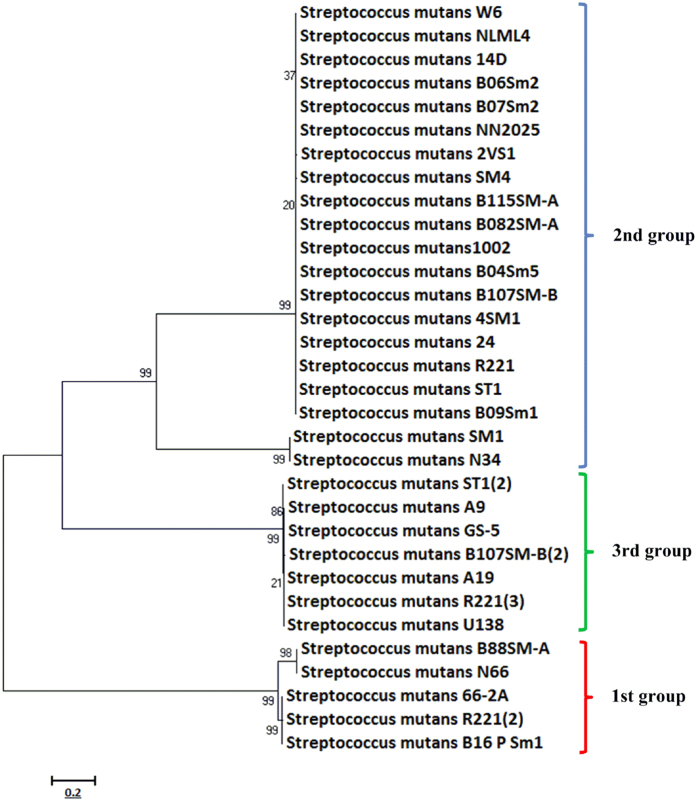
Evolutionary relationships among peptidase C39 domain-containing proteins in lantibiotic gene clusters from *S. mutans*. The phylogenetic tree was made by MEGA 7.0 using the Maximum likelihood method and with 1000 bootstrap replications for each branch. The bootstrap value of nodes is indicated. Each taxon is composed of a single strain and the major clades are marked with different colors (blue, red and green) and corresponding family classification. The number in brackets following the strain names indicates the number of lantibiotic gene clusters identified if more than one.

Previous phylogenetic analysis of cyanobacteria proposed that the C39 domain-containing ABC transporters could reveal the evolutionary history of different C39 domain-containing transporters and their corresponding gene clusters^36^. We employed a similar analysis to dissect the phylogenetic relationship among the lantibiotic gene clusters. The independent C39 protease (encoding by gene P) from the first group was artificially joined with the following ABC transporter (encoding by gene T) and aligned with the C39 domain-containing ABC transporters from the other two groups. The constructed phylogenetic tree (Fig. 6) reveals three distinct groups that correspond well with the above classification based on gene organization, with the first group more distantly related than the other two.

### Unmodified bacteriocin gene clusters in *S. mutans*

**Figure 7:**
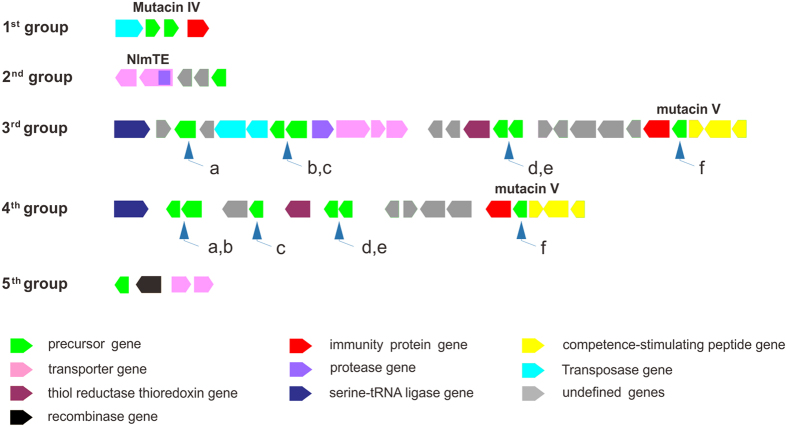
Composition of unmodified bacteriocin gene clusters in *S. mutans*. Functions of all genes are indicated by different colors. NlmTE, mutacin IV and mutacin V labels are located above the genes encoding homologs of them.

Anti-SMASH and BAGEL analysis of the 169 *S. mutans* genomes identified 179 gene clusters encoding unmodified bacteriocins that were classified into five groups based on gene organization (Fig. 7 and Supplementary Table SII). The first group contains 63 members, each containing two adjacent precursor genes flanked by an upstream transpose gene and a downstream immunity protein gene^37^. Mutacin IV from the unsequenced strain *S. mutans* UA140 represents the best-studied example of this group, and this putative two-peptide (mutacin IV-1 and IV-2) bacteriocin has potent antibiotic activity against the mitis group of oral *streptococci*^23^. Analysis of precursor genes of this group revealed that most (>85%) encode identical precursor peptides (named as mutacin IV-1 and mutacin IV-2 in Supplementary Figure SI), while the others have minor variants in their leader peptides, indicating that mutacin IV biosynthesis in *S. mutans* is highly conserved (data not shown).

**Figure 8:**
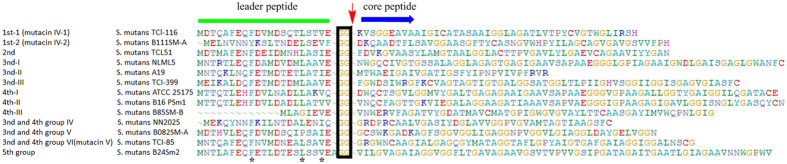
Alignment of unmodified bacteriocin precursors in *S. mutans*. The names of representative bacteriocin precursors and their host strains on the left. The conserved “GG” motif is boxed; the cleavage site is indicated by an arrow; conserved leader peptide amino acids are marked with asterisks.

Twenty-four unmodified bacteriocin gene clusters belonging to the 2^nd^ group were identified that each possessed a simple and invariant gene organization of one precursor gene, two unknown genes and two ABC transporter genes with one containing a protease domain (Fig. 7). The two ABC transporters in *S. mutans* UA159, named NlmTE, were found to transport mutacin IV out of the cell^38^. Interestingly, no protease gene was identified in the 1^st^ group unmodified bacteriocin gene clusters, and the high similarity between leader peptides of the precursors in the first and second groups (Fig. 8) suggests that the two groups might share transporters like NlmTE in strains such as *S. mutans* UA159 that harbor both groups.

There are 73 unmodified bacteriocin gene clusters belonging to the third group, which has the most complicated gene organization with six precursor genes, three transporter genes, one immunity gene, one protease gene and several other genes with varied functions, such as serine-tRNA ligase, transposase and unknown functions (Fig. 7). The only knowledge about this group of clusters was from *S. mutans* UA159, in which deletion of the “ f ” precursor gene (SMU.1914c) changed the strain’s antibacterial profile, and the unidentified product of the precursor “ f ” gene in *S. mutans* UA159 was called mutacin V^39^.

The fourth group of unmodified bacteriocin gene cluster contains 16 members, which appears to be a simplified version of the third group gene cluster. Thirteen genes comprising the left half of the third group cluster between the serine-tRNA ligase gene and the thioredoxin gene are replaced by three new precursor genes and a gene with unknown function in the fourth group clusters. The remaining precursors (precursor“d”, “e”, and “f”) on the right side of the fourth group clusters were almost identical to those in the third group, implying a close evolutionary relationship between these two groups of unmodified bacteriocin gene clusters.

The fifth group consists of three unmodified bacteriocin gene clusters with a precursor gene and two transporter genes. Interestingly, an uncommon gene that encodes a putative recombinase is located next to the precursor gene, indicating that the precursor gene may have originated from another location in the genome or from foreign DNA.

An alignment of representative precursors of unmodified bacteriocins (Fig. 8) reveals conserved leader peptides that harbor a “GG” motif that is usually located before the proteolytic cleavage site. On the contrary, the diversity of core peptide sequences demonstrates strong potential for the discovery of new bacteriocins from *S. mutans*.

In addition to the unmodified bacteriocin gene clusters mentioned above, we also found 173 putative bacteriocin precursor genes in *S. mutans,* which even contain 20 mutacin VI homologous precursor genes. Because no related genes (e.g. protease, immunity and transporter genes) could be identified nearby, we treated them as orphan precursor genes that may encode novel bacteriocins and summarized them with the representatives in Supplementary Figure SII.

## Discussion

We have identified 355 natural product gene clusters from the genomes of 169 strains of the human oral microbe *S. mutans*, and 39% of these strains contained more than 2 clusters. Since only five genomes (until November 19^th^, 2015) were sequenced in complete level with the others in draft, the number of natural product gene clusters is undoubtedly much higher. Although bacteria with small genomes have been considered a poor source of PKS- and NRPS-synthesized natural products^13^,^14^, our discovery of 144 PKS, NRPS and hybrid PKS/NRPS clusters and 211 RiPPs clusters in the 169 strains suggests bacteria with small genome sizes like *S. mutans* may represent important and overlooked sources of natural products.

That *S. mutans* houses so many NRPS and hybrid PKS/NRPS secondary metabolite gene clusters in its small genome is surprising and suggests *S. mutans* may dispatch a battery of antibiotic weapons to fight, communicate and thrive in its particular niche. The relative paucity of A-T-C domains encoded by other species of oral *Streptococcus*, such as *Streptococcus oligofermentans* (Supplementary Table SIV), suggests that the environment of dental caries has selected for a robust genetic complement of natural product capacity in *S. mutans*. Similarly, the wide spread of bacteriocin gene clusters in *S. mutans* may also due to the selective pressure from the complicated oral environment. It has been shown that *S. mutans* apply some mutacins to build up competitive advantage in biofilm formation and in colonization in new host^40^,^41^. In addition, a number of mutacins can influence the competence capacity of *S. mutans*, e.g. the small peptide ComC produced by an unmodified bacteriocin gene cluster can induce *S. mutans* UA150 to a high competence state efficiently; and, an apparent lower transformation efficiency was observed in the mutacin IV or V mutant strains^42^,^43^. Therefore, mutacins might be functional not only as antibiotics against the competitive microbes, but also as genetic tools prompting the DNA exchange frequency of *S. mutans*, which all render a better fitness of this species in its oral habitat.

Historically, microbial natural product chemists have intensively investigated microbes from soil, water and other natural environments, but have generally overlooked the microorganisms hosted by our own bodies. Recent human microbiome research has not only highlighted the importance of the human microbiome to health, but has also revealed a new scientific frontier of deciphering the precise chemical language of communication among and between microbes and the human host^44^,^45^,^46^,^47^. Because secondary metabolites frequently exhibit antimicrobial and signaling activities, characterization of compounds from human commensal or pathogenic microbes can enlighten our understanding of the host-microbe relationship. As a causative agent of human tooth caries, understanding *S. mutans* secondary metabolism can help us understand the biology of tooth decay and provide therapeutic targets for future preventative treatments. The discovery of a rich source of natural product gene clusters from the 169 genomes of *S. mutans* from human caries sets the stage for isolating these compounds and elucidating their biological roles. Significantly, compounds from *S. mutans* are already active against gram positive pathogenic bacteria (e.g. mutacin 1140) and eukaryotic pathogens (e.g. mutanobactins), and the abundance of natural product gene clusters in *S. mutans* genomes predicts that many more fascinating compounds await discovery.

## Methods

### Data and strains

Complete and draft genomes of 169 *S. mutans* were collected from public genome database of NCBI (until November 19^th^, 2015) and saved in FASTA format. Their detailed information is listed in Supplementary Table SIII. The protein information of transporters with protease domains or with flanking protease genes from lantibiotic gene clusters was provided in Supplementary Table SV.

### Detection of putative natural product gene clusters

AntiSMASH (Antibiotics & Secondary Metabolite Analysis SHell) was used with default settings to identify NRPS and PKS gene clusters^48^. Functional domain prediction was performed with NRPSpredictor2 and NaPDoS (Natural Product Domain Seeker)^49^,^50^. Annotations were made using Conserved Domain search and BLASTp (Basic Local Alignment Search Tool)^51^. The borders of these gene clusters were manually refined by checking synteny among protein sequences within the automatically predicted gene clusters from antiSMASH.

RiPPs precursors were identified through a combination of antiSMASH and BAGEL^52^. The results from both tools were compared, and only those gene clusters positive in both methods were analyzed in this study. The lantibiotic gene clusters were refined by visualizing all genes required for lantibiotic peptide biosynthesis. The unmodified bacteriocin gene clusters were identified if precursor-encoding genes were located together with transporters, immunity proteins or proteases.

### Phylogenetic study

The phylogenetic study was conducted on protein sequences of transporters having a protease domain or a protease gene in the lantibiotic gene clusters using MEGA7.0 software employing the Maximum likelihood method. The protein sequences were retrieved from NCBI in FASTA format and aligned by clustalW in MEGA7.0.

## Additional Information

**How to cite this article**: Liu, L. *et al.* Genome mining unveils widespread natural product biosynthetic capacity in human oral microbe *Streptococcus mutans*. *Sci. Rep.* **6**, 37479; doi: 10.1038/srep37479 (2016).

**Publisher’s note:** Springer Nature remains neutral with regard to jurisdictional claims in published maps and institutional affiliations.

## Supplementary Material

Supplementary Information
