# Supplementary material for: Genome mining unveils widespread natural product biosynthetic capacity in human oral microbe Streptococcus mutans
Source: Sci Rep. 2016 Nov 21;6:37479. doi: 10.1038/srep37479 (PMC5116633; doi:10.1038/srep37479)
Supplement: Supplementary Information [file srep37479-s1.pdf]

Genome mining unveils widespread natural product biosynthetic capacity in human oral microbe *Streptococcus mutans*

Liwei Liu 1#, Tingting Hao 1,2#, Zhoujie Xie 1, Geoff P. Horsman 3, Yihua Chen 1,2*

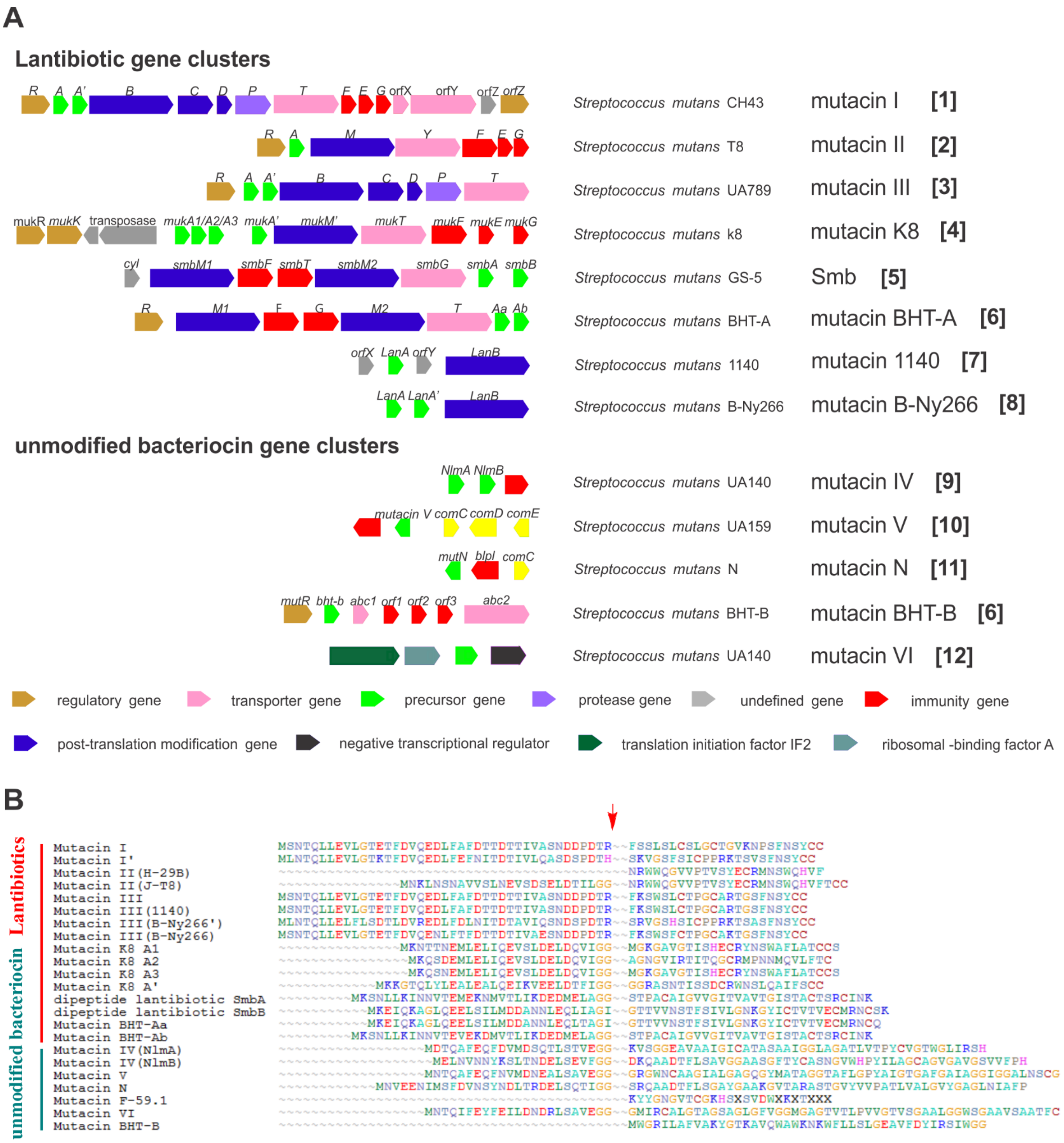

Figure SI. Composition of reported biosynthetic gene clusters of lantibiotics and unmodified bacteriocins in *S. mutans*. A: The gene clusters were depicted in details. The functional proteins are indicated in colors below, and gene names are marked above each arrow. The host strains with corresponding products are indicated behind each gene cluster. **B:** The identified lantibiotics peptide and unmodified bacteriocins are aligned manually with Bioedit. The lantibiotics are indicated with red color, while the non lantibiotic (unmodified bacteriocins) are shown in green color. The core peptides are divided from leader peptide with blank space.[1-12]

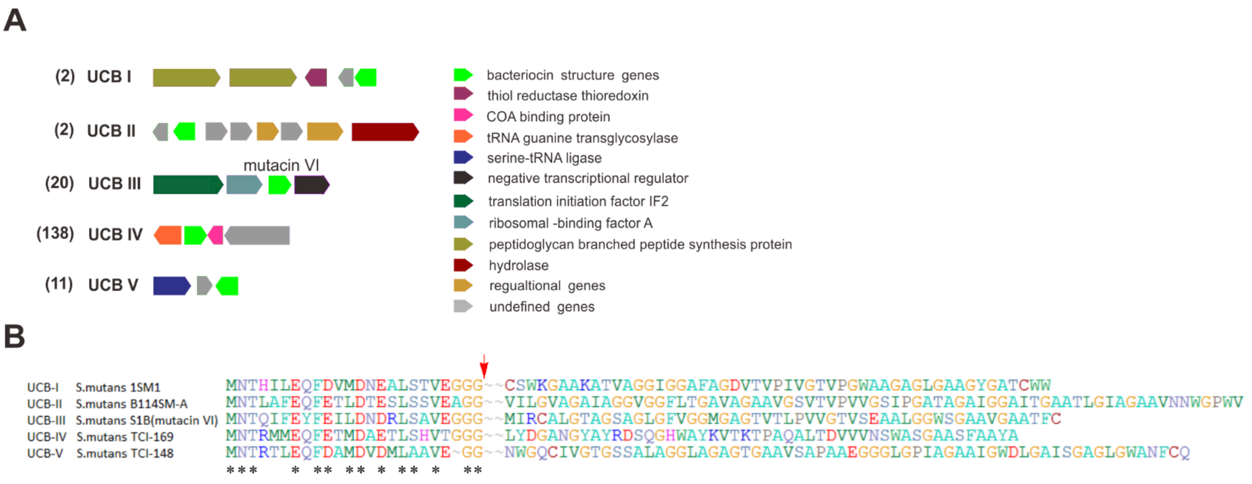

Figure SII. Composition of the orphan bacteriocins "clusters" in *S. mutans* (A) and alignment of their representative precursors (B). A: Of 173 precursors of orphan bacteriocins are discovered from 169 *S. mutans* genomes. Genes around the orphan precursor genes are also depicted as a cluster. The number of each group of "cluster" is marked at left. The functional proteins are indicated with colorful arrows on the right panel. **B**: The representative precursors from each group are aligned manually using MEGA7. The conserved amino acids in their leader peptides are marked with asterisks, and putative cleavage site is pointed out with a red arrow.

Table SI. The abundance of NRPS, PKS and hybrid PKS/NRPS gene clusters among 169 *S. mutans*.

Gene cluster classification	Number	groups					
**NRPS**	1			-			
**PKS**	7			-			
**Hybrid**	136	I	II	III	IV	V	VI
		58	22	29	4	4	19
**Total**	144			-			

Table SII. The abundace of lantibiotics and unmodified bacteriocin gene clusters and their precursors in 169 *S. mutans*.

Group	NO. of gene cluster	NO. of precursor	known gene clusters
**Lan 1st**	5	8	Mutacin I, III, 1140, B-Ny266
**Lan 2nd**	20	34	Mutacin K8
**Lan 3rd**	7	14	Mutacin Smb/BHT-A
**Total (LAN)**	32	56	-
**U.Bac 1st group**	63	118	Mutacin IV
**U.Bac 2nd group**	24	24	-
**U.Bac 3rd group**	73	335	Mutacin IV
**U.Bac 4th group**	16	79	Mutacin IV
**U.Bac 5th group**	3	3	-
**Total (U.Bac)**	179	559	-
**Total**	211	615	-

LAN: lantibiotic; U.Bac: unmodified bacteriocin.

Table SIII. Distribution of natural product gene clusters among 169 *S. mutans* strains and detail information of 169 *S. mutans* genomes.

Organism/Strain Name	NRPS	PKS	Hybrid NRPS/PKS	Lantibiotics	Unmodified bacteriocins	Orphan bacteriocin precursors	Access Number	Size (Mb)	GC%	Scaffolds	Level
Streptococcus mutans UA159			A		A, B, C	D	AE014133.2	2.03293	36.8	1	Complete Genome
Streptococcus mutans NN2025			B,C	B	B, C	D	AP010655.1	2.01359	36.8	1	Complete Genome
Streptococcus mutans GS-5			A	C	A, B, D	D	CP003686.1	2.02709	36.8	1	Complete Genome
Streptococcus mutans LJ23			F			C,D	AP012336.1	2.01563	37.1	1	Complete Genome
Streptococcus mutans UA159-FR			A		A, C	D	CP007016.1	2.03169	36.8	1	Complete Genome
Streptococcus mutans TCI-101						D	AGKF00000000.1	1.81619	37	141	Contig
Streptococcus mutans TCI-109							AGKG00000000.1	1.80167	37	146	Contig
Streptococcus mutans TCI-11							AGKH00000000.1	1.79992	37	141	Contig
Streptococcus mutans TCI-110					A, C	D	AGKI00000000.1	1.80911	37	135	Contig
Streptococcus mutans TCI-116			A		A, B, C	D	AGKJ00000000.1	1.92969	36.7	60	Contig
Streptococcus mutans TCI-120							AGKK00000000.1	1.7769	37	159	Contig
Streptococcus mutans TCI-123			A		A	D	AGKL00000000.1	1.8905	36.8	109	Contig
Streptococcus mutans TCI-125			A		A	D	AGKM00000000.1	1.86169	36.8	96	Contig
Streptococcus mutans TCI-138						D	AGKN00000000.1	1.80508	37	131	Contig
Streptococcus mutans TCI-143							AGKO00000000.1	1.77631	37	157	Contig
Streptococcus mutans TCI-145			A		A	D	AGKP00000000.1	1.87779	36.8	99	Contig
Streptococcus mutans TCI-146					C	D	AGKE00000000.1	1.78549	37	143	Contig
Streptococcus mutans TCI-148						D,E	AGKQ00000000.1	1.81941	37	120	Contig
Streptococcus mutans TCI-149					C	D	AGKR00000000.1	1.82264	37	117	Contig
Streptococcus mutans TCI-151					C	D	AGKS00000000.1	1.82047	37	121	Contig
Streptococcus mutans TCI-152			A		A, C	D	AGKT00000000.1	1.8514	36.9	101	Contig
Streptococcus mutans TCI-153			A		A, B, C	D	AGKU00000000.1	1.92765	36.7	55	Contig

Streptococcus mutans TCI-154			D,E	AGKV00000000.1	1.81895	37	120	Contig
Streptococcus mutans TCI-162			D,E	AGKW00000000.1	1.81787	37	130	Contig
Streptococcus mutans TCI-163	A	A, C		AGKX00000000.1	1.89788	36.8	92	Contig
Streptococcus mutans TCI-164		C		AGKY00000000.1	1.8292	37	115	Contig
Streptococcus mutans TCI-169	A	A, B, C	D	AGKZ00000000.1	1.92777	36.7	59	Contig
Streptococcus mutans TCI-170	A	C	D	AGLA00000000.1	1.89949	36.8	86	Contig
Streptococcus mutans TCI-173	A	A, C	D	AGLB00000000.1	1.87268	36.8	112	Contig
Streptococcus mutans TCI-176		A, C	D	AGLC00000000.1	1.79475	37	128	Contig
Streptococcus mutans TCI-177	A	A	D	AGLD00000000.1	1.88295	36.8	110	Contig
Streptococcus mutans TCI-179			D,E	AGLE00000000.1	1.8182	37	126	Contig
Streptococcus mutans TCI-187		A		AGLF00000000.1	1.76556	37	127	Contig
Streptococcus mutans TCI-191		C		AGLG00000000.1	1.83201	37	120	Contig
Streptococcus mutans TCI-196	A			AGLH00000000.1	1.89819	36.8	90	Contig
Streptococcus mutans TCI-201				AGLI00000000.1	1.77085	37	146	Contig
Streptococcus mutans TCI-202			D	AGLJ00000000.1	1.82031	37	115	Contig
Streptococcus mutans TCI-204		A		AGLK00000000.1	1.84716	37	136	Contig
Streptococcus mutans TCI-210		A, C	D	AGLL00000000.1	1.80935	37	135	Contig
Streptococcus mutans TCI-212		A	D	AGLM00000000.1	1.77143	37	194	Contig
Streptococcus mutans TCI-218		A		AGLN00000000.1	1.78937	37	136	Contig
Streptococcus mutans TCI-219			D	AGLO00000000.1	1.80927	37	131	Contig
Streptococcus mutans TCI-220				AGLP00000000.1	1.79064	37	145	Contig
Streptococcus mutans TCI-222	A	A	D	AGLQ00000000.1	1.86163	36.8	101	Contig
Streptococcus mutans TCI-223			D	AGLR00000000.1	1.80957	37	134	Contig
Streptococcus mutans TCI-224				AGLS00000000.1	1.79774	37	133	Contig
Streptococcus mutans TCI-227			D	AGLT00000000.1	1.8029	37	130	Contig
Streptococcus mutans TCI-228	A	A	D	AGLU00000000.1	1.86	36.9	109	Contig

Streptococcus mutans TCI-234	A	B	D	AGLV00000000.1	1.85493	36.8	106	Contig
Streptococcus mutans TCI-239	A			AGLW00000000.1	1.89822	36.8	86	Contig
Streptococcus mutans TCI-242	A	A, B, C	D	AGLX00000000.1	1.9144	36.8	61	Contig
Streptococcus mutans TCI-243	A	A, B, C	D	AGLY00000000.1	1.9147	36.8	66	Contig
Streptococcus mutans TCI-244				AGLZ00000000.1	1.77114	37	143	Contig
Streptococcus mutans TCI-249	A	A	D	AGMA00000000.1	1.87941	36.8	98	Contig
Streptococcus mutans TCI-256	A	A, C		AGMB00000000.1	1.88111	36.8	110	Contig
Streptococcus mutans TCI-260			D	AGMC00000000.1	1.799	37	141	Contig
Streptococcus mutans TCI-264		C	D	AGMD00000000.1	1.8246	37	137	Contig
Streptococcus mutans TCI-267			D,E	AGME00000000.1	1.81974	37	127	Contig
Streptococcus mutans TCI-268	A	A	D	AGMF00000000.1	1.85568	36.9	92	Contig
Streptococcus mutans TCI-278	A	A	D	AGMG00000000.1	1.85634	36.9	98	Contig
Streptococcus mutans TCI-279			D	AGMH00000000.1	1.80982	37	136	Contig
Streptococcus mutans TCI-280			D	AGMI00000000.1	1.81879	37	119	Contig
Streptococcus mutans TCI-289			D	AGMJ00000000.1	1.7982	37	140	Contig
Streptococcus mutans TCI-292			D	AGMK00000000.1	1.81088	37	137	Contig
Streptococcus mutans TCI-294	A	A	D	AGML00000000.1	1.89133	36.8	109	Contig
Streptococcus mutans TCI-298			D	AGMM00000000.1	1.7435	37	136	Contig
Streptococcus mutans TCI-30	A	A, C	D	AGMN00000000.1	1.88003	36.8	103	Contig
Streptococcus mutans TCI-399	A	A, B, C	D	AGMO00000000.1	1.93573	36.7	53	Contig
Streptococcus mutans TCI-400	A	A, B, C	D	AGMP00000000.1	1.86962	36.8	93	Contig
Streptococcus mutans TCI-51	A	A, B, C	D	AGMQ00000000.1	1.93689	36.7	57	Contig
Streptococcus mutans TCI-62			D	AGMR00000000.1	1.80483	37	135	Contig
Streptococcus mutans TCI-70	A	A		AGMS00000000.1	1.8556	36.8	109	Contig
Streptococcus mutans TCI-75	A	A	D	AGMT00000000.1	1.87948	36.8	108	Contig
Streptococcus mutans TCI-78	A	A	D	AGMU00000000.1	1.88309	36.8	110	Contig

Streptococcus mutans TCI-82		A		A, B, C	D	AGMV00000000.1	1.94123	36.7	48	Contig
Streptococcus mutans TCI-85		A		A, B, C	D	AGMW00000000.1	1.92185	36.8	64	Contig
Streptococcus mutans TCI-86						AGMX00000000.1	1.82001	37	128	Contig
Streptococcus mutans TCI-92		A		A, B, C	D	AGMY00000000.1	1.94139	36.7	49	Contig
Streptococcus mutans TCI-96				A	E	AGMZ00000000.1	1.80055	37	121	Contig
Streptococcus mutans TCI-99				A, C	D	AGNA00000000.1	1.81479	37	132	Contig
Streptococcus mutans S1B		C		C	C	AHRC00000000.1	2.00636	36.8	132	Contig
Streptococcus mutans SA41		A		C	D	AHRE00000000.1	1.95232	36.8	104	Contig
Streptococcus mutans SF12				C	C,D	AHRF00000000.1	1.92589	36.9	107	Contig
Streptococcus mutans R221			A,B,C	C	D	AHRG00000000.1	1.98372	36.8	109	Contig
Streptococcus mutans M230		C		C		AHRH00000000.1	1.91745	36.9	139	Contig
Streptococcus mutans 15JP3					D	AHRJ00000000.1	1.93297	36.9	87	Contig
Streptococcus mutans 4SM1	1	F	B	C	C,D	AHRL00000000.1	2.00534	36.7	97	Contig
Streptococcus mutans 2ST1		A			D	AHRN00000000.1	1.96556	36.7	111	Contig
Streptococcus mutans 4VF1	1	F			D	AHRQ00000000.1	2.00873	36.6	127	Contig
Streptococcus mutans 15VF2	1	F		A, C	D	AHRR00000000.1	2.02167	36.6	117	Contig
Streptococcus mutans 11VS1		C		D	D	AHRT00000000.1	1.86196	36.6	155	Contig
Streptococcus mutans 5SM3		D,E		D	D	AHRU00000000.1	1.94364	36.8	86	Contig
Streptococcus mutans NFSM2		A		C	D	AHRV00000000.1	1.99098	36.6	115	Contig
Streptococcus mutans A9			C	A	D	AHRX00000000.1	1.86849	36.8	105	Contig
Streptococcus mutans N29		D,E		D	B,C,D	AHRY00000000.1	1.96551	36.8	110	Contig
Streptococcus mutans NMT4863		D,E		C	C,D	AHRZ00000000.1	1.9266	36.8	97	Contig
Streptococcus mutans T4		A			D	AHSE00000000.1	1.96605	36.7	113	Contig
Streptococcus mutans NFSM1		C		C	A,C,D	AHSG00000000.1	2.00211	36.9	131	Contig
Streptococcus mutans NV1996		F		C	D	AHSN00000000.1	2.01516	36.8	130	Contig
Streptococcus mutans SF14		F		C	C,D	AHSQ00000000.1	1.94479	36.8	97	Contig

Streptococcus mutans SM6		B,C		C	D	AHSR00000000.1	1.96047	36.8	112	Contig
Streptococcus mutans U2A		F			D	AHSU00000000.1	2.09943	36.8	163	Contig
Streptococcus mutans NLML8		F			D	AHSV00000000.1	1.96315	36.8	135	Contig
Streptococcus mutans 14D		B,C	B		D	AHSY00000000.1	1.9691	36.7	102	Contig
Streptococcus mutans 21		A			D	AHSZ00000000.1	1.99007	36.7	120	Contig
Streptococcus mutans B		B,C			D	AHTB00000000.1	1.94812	36.7	111	Contig
Streptococcus mutans SM1		F	B		D	AHTD00000000.1	1.99493	36.7	114	Contig
Streptococcus mutans 8ID3				C	D	AHRB00000000.1	1.90874	36.9	100	Contig
Streptococcus mutans SA38	1	F		C	C,D	AHRD00000000.1	1.99289	36.7	91	Contig
Streptococcus mutans OMZ175		F			C,D	AHRI00000000.1	2.00323	36.8	130	Contig
Streptococcus mutans 1SM1	1	F		A	A,C,D	AHRK00000000.1	2.06848	36.6	102	Contig
Streptococcus mutans 3SN1		C		C	D	AHRM00000000.1	2.03435	36.8	142	Contig
Streptococcus mutans 11A1					D,E	AHRO00000000.1	1.92165	36.9	111	Contig
Streptococcus mutans 11SSST2		A		C		AHRP00000000.1	1.96961	36.7	105	Contig
Streptococcus mutans 2VS1		B,C	B		D	AHRS00000000.1	1.97001	36.6	98	Contig
Streptococcus mutans NVAB					D	AHRW00000000.1	1.94417	36.8	102	Contig
Streptococcus mutans A19		A	C	C		AHSA00000000.1	1.98402	36.7	108	Contig
Streptococcus mutans U138		A	C	C		AHSB00000000.1	1.99196	36.7	153	Contig
Streptococcus mutans G123		B,C			C,D	AHSC00000000.1	1.97704	36.7	122	Contig
Streptococcus mutans M21		B,C		C	D	AHSD00000000.1	1.96197	36.8	106	Contig
Streptococcus mutans N34		F	B	C	C,D	AHSF00000000.1	1.96203	36.8	102	Contig
Streptococcus mutans NLML4		B,C	B		D	AHSH00000000.1	1.96863	36.7	116	Contig
Streptococcus mutans NLML5		F		A, C	D	AHSI00000000.1	1.94139	36.8	105	Contig
Streptococcus mutans NLML9		B,C			D	AHSJ00000000.1	1.99601	36.7	99	Contig
Streptococcus mutans M2A		B		A, C	C,D	AHSK00000000.1	1.93024	36.9	115	Contig
Streptococcus mutans N3209		A			D	AHSL00000000.1	1.9534	36.7	113	Contig

Streptococcus mutans N66			A	C	D,E	AHSM00000000.1	2.00604	36.8	113	Contig
Streptococcus mutans W6		B,C	B		D	AHSO00000000.1	1.99136	36.6	114	Contig
Streptococcus mutans SF1		C		C		AHSP00000000.1	2.08142	36.8	185	Contig
Streptococcus mutans ST1			B,C	C	D	AHSS00000000.1	1.98046	36.7	131	Contig
Streptococcus mutans ST6		C		C	D	AHST00000000.1	1.92585	36.9	121	Contig
Streptococcus mutans NLML1	1	F			D	AHSW00000000.1	2.03603	36.6	139	Contig
Streptococcus mutans 1ID3		F		A, C	D	AHSX00000000.1	1.87237	36.9	90	Contig
Streptococcus mutans 66-2A		F	A	C	D	AHTA00000000.1	1.95301	36.9	113	Contig
Streptococcus mutans SM4		C	B	C		AHTC00000000.1	1.97758	36.9	152	Contig
Streptococcus mutans 24		B,C	B	A	C,D	AHTE00000000.1	2.01368	36.7	119	Contig
Streptococcus mutans U2B					D,E	AGWE00000000.1	1.96014	36.9	103	Contig
Streptococcus mutans 5DC8		A		A, B, C	D	AOBX00000000.1	2.01095	36.9	9	Contig
Streptococcus mutans KK21				A, B	D	AOBY00000000.1	2.03459	36.8	2	Contig
Streptococcus mutans KK23		B,C		A, C	C	AOBZ00000000.1	1.9762	36.7	38	Contig
Streptococcus mutans AC4446		B,C			D	AOCA00000000.1	2.0037	36.9	42	Contig
Streptococcus mutans ATCC 25175		A		A, B, D	D	AOCB00000000.1	1.99967	36.9	10	Contig
Streptococcus mutans NCTC 11060		F		A, C		AOCC00000000.1	2.02131	37	36	Contig
Streptococcus mutans DSM 20523		A		A, B, D	D	AQWT00000000.1	1.98877	36.7	20	Contig
Streptococcus mutans PKUSS-HG01		A		D	D	AXSW00000000.1	2.00261	36.6	12	Contig
Streptococcus mutans PKUSS-LG01		A		A, C		AXSX00000000.1	2.00139	36.7	14	Contig
Streptococcus mutans B05Sm11		B		A, C	C,D	ALYO00000000.1	2.03637	37	53	Contig
Streptococcus mutans B13Sm1		A		A	D	ALYP00000000.1	2.15193	36.8	46	Contig
Streptococcus mutans B12Sm1		C		B, C	D,E	ALYQ00000000.1	2.17358	36.9	91	Contig
Streptococcus mutans B084SM-A				A ,C	D	ALYR00000000.1	2.04821	36.9	61	Contig
Streptococcus mutans B107SM-B		B,C	B,C	C	D	ALYS00000000.1	2.10271	36.8	57	Contig
Streptococcus mutans B07Sm2			B	B	D	ALYT00000000.1	2.22226	36.8	49	Contig

Streptococcus mutans B09Sm1			C	B		D,E	ALYU00000000.1	2.15448	37	106	Contig
Streptococcus mutans B24Sm2	1	1	F		A,E	C,D	ALYV00000000.1	2.28914	36.8	63	Contig
Streptococcus mutans B102SM-B			A		A, D	D	ALYW00000000.1	2.0952	36.8	104	Contig
Streptococcus mutans B112SM-A			D,E		C	D(2)	ALYX00000000.1	2.25916	37.1	44	Contig
Streptococcus mutans B04Sm5			B,C	B	A, C	C,D	ALYY00000000.1	2.07762	37	81	Contig
Streptococcus mutans B082SM-A			B,C	B	C	D	ALYZ00000000.1	2.12675	37	53	Contig
Streptococcus mutans B06Sm2			B,C	B	C	D	ALZA00000000.1	2.21455	36.9	65	Contig
Streptococcus mutans B85SM-B			A		B, D	D	ALZB00000000.1	2.10192	36.9	46	Contig
Streptococcus mutans B88SM-A			A	A	A, D	D	ALZC00000000.1	2.06195	36.7	75	Contig
Streptococcus mutans str. B16 P Sr			A	A	D		ALZD00000000.1	2.32985	36.9	58	Contig
Streptococcus mutans B23Sm1			C		D	C,D	ALZE00000000.1	2.09541	37.1	87	Contig
Streptococcus mutans B111SM-A			A		A, B, D	D	ALZF00000000.1	2.12499	36.9	39	Contig
Streptococcus mutans B114SM-A			A		D	B,D	ALZG00000000.1	2.11043	37	53	Contig
Streptococcus mutans B115SM-A			B	B	C	D	ALZH00000000.1	2.28358	36.9	49	Contig
Streptococcus mutans 1006_SMUT			B,C		D,E	D	JWGO00000000.1	2.02615	36.7	62	Contig
Streptococcus mutans 1002_SMUT			B	B	D,E		JWGS00000000.1	2.03597	36.8	49	Contig
Streptococcus mutans 503_SMUT			A		B	D	JVEO00000000.1	2.00755	36.8	30	Scaffold
Total	1	7	136	32	179	173					

NRPS/PKS gene clusters: A, group I; B, group II; C, group III; D, group IV; E, group V; F, group VI

Lantibiotic gene clusters: A, 1st group; B, 2nd group; C, 3rd group

Unmodified bacteriocin gene cluster: A, 1st group; B, 2nd group; C, 3rd group; D, 4th group; E, 5th group

Orphan bacteriocin precursor gene: A, 1st group; B, 2nd group; C, 3rd group; D, 4th group; E, 5th group

Table SIV. The A-T-C module and KS-AT-T module distributed in *Streptococcus* spp. and *S. mutans* were screened with Conserved Domain Architecture Retrieval Tool (CDART).

	**A-T-C module**	**KS-AT-T module**	**Number of str**
Streptococcus **s**	2783	4481	10038
S. mutans	338	471	169
S. oligofermenta	3	2	7

Table SV. Transporters with C39 protease domains or with flanking protease genes from the 32 identified lantibiotic gene clusters of *S. mutans*.

Strain name	Gi NO.	Function	Group
Streptococcus mutans R221	>gi|449260079|gb|EMC57589.1| >gi|449260080|gb|EMC57590.1|	peptidase<br>ABC transporter	1st
Streptococcus mutans B16 P Sm1	> gi|575868072|gb|ALZD01000009.1|	peptidase<br>ABC transporter	1st
Streptococcus mutans B88SM-A	>gi|575868113|gb|ALZC01000020.1|	peptidase<br>ABC transporter	1st
Streptococcus mutans 66-2A	>gi|449240026|gb|EMC38722.1| >gi|449240025|gb|EMC38721.1|	peptidase<br>ABC transporter	1st
Streptococcus mutans N66	>gi|449214895|gb|EMC15127.1| >gi|449214896|gb|EMC15128.1|	peptidase<br>ABC transporter	1st
Streptococcus mutans NN2025	>gi|254996752|dbj|BAH87353.1|	ScnT-like protein	2nd
Streptococcus mutans B115SM-A	>gi|575868301|gb|ALZH01000042.1|	ScnT-like protein	2nd
Streptococcus mutans B082SM-A	>gi|575867712|gb|ALYZ01000051.1|	ScnT-like protein	2nd
Streptococcus mutans B07Sm2	>gi|575867344|gb|ALYT01000028.1|	ScnT-like protein	2nd
Streptococcus mutans1002	>gi|876133508|gb|JWGS01000045.1|	ScnT-like protein	2nd
Streptococcus mutans B06Sm2	>gi|575867836|gb|ALZA01000034.1|	hypothetical protein	2nd
Streptococcus mutans B04Sm5	>gi|575867870|gb|ALYY01000027.1|	hypothetical protein	2nd
Streptococcus mutans SM4	>gi|449246489|gb|EMC44792.1|	hypothetical protein	2nd
Streptococcus mutans 14D	>gi|449233821|gb|EMC32868.1|	hypothetical protein	2nd
Streptococcus mutans SM1	>gi|449243113|gb|EMC41575.1|	NukT	2nd
Streptococcus mutans B107SM-B	>gi|575867223|gb|ALYS01000043.1|	hypothetical protein	2nd
Streptococcus mutans W6	>gi|449218748|gb|EMC18747.1|	hypothetical protein	2nd
Streptococcus mutans NLML4	>gi|449205304|gb|EMC06056.1|	hypothetical protein	2nd
Streptococcus mutans 2VS1	>gi|449171745|gb|EMB74393.1|	hypothetical protein	2nd
Streptococcus mutans ST1	>gi|449227128|gb|EMC26572.1|	hypothetical protein	2nd
Streptococcus mutans B09Sm1	>gi|575867522|gb|ALYU01000054.1|	hypothetical protein	2nd
Streptococcus mutans N34	>gi|449201723|gb|EMC02705.1|	NukT	2nd
Streptococcus mutans 4SM1	>gi|449160736|gb|EMB63978.1|	hypothetical protein	2nd
Streptococcus mutans 24	>gi|449246914|gb|EMC45208.1|	hypothetical protein	2nd
Streptococcus mutans R221	>gi|449261139|gb|EMC58623.1|	hypothetical protein	2nd
Streptococcus mutans ST1	>gi|449226656|gb|EMC26165.1|	hypothetical protein	3rd
Streptococcus mutans A9	>gi|449184765|gb|EMB86684.1|	hypothetical protein	3rd
Streptococcus mutans B107SM-B	>gi|575867272|gb|ALYS01000025.1|	hypothetical protein	3rd
Streptococcus mutans A19	>gi|449188680|gb|EMB90381.1|	hypothetical protein	3rd
Streptococcus mutans R221	>gi|449261228|gb|EMC58709.1|	hypothetical protein	3rd
Streptococcus mutans U138	>gi|449194607|gb|EMB95959.1|	hypothetical protein	3rd
Streptococcus mutans GS-5	>gi|392604049|gb|AFM82213.1|	Toxin RTX-I translocation<br>ATP-binding protein	3rd

Group: based on lantibiotic gene cluster classification.

Methods

The fast-screening of modular genes (NRPS, PKS or hybrid) in *Streptococcus spp*.

The amino acid sequence of SMU_1341c in *S. mutans* UA159 (AAN59014.1) and SMU56_08633 in *S. mutans* N29 (EMB85481.1) were used to search for A-T-C module for NRPS and KS-AT-T module for PKS from Entrez Protein database limited to genome-annotated *Streptococcus spp*. from NCBI (10,038 strains till Apr. 18th 2016) with CDART. In order to test feasibility of this method, protein abyB3 (AEK75504.1) from *Verrucosispora maris* AB-18-032and aptB (ACZ55943.1) from *Anabaena sp*. 90 were applied in the same screening. The results of twice tests were identical.

Reference

1. Qi, F.X., Chen, P., Caufield, P.W. Purification and biochemical characterization of mutacin I from the group I strain of *Streptococcus mutans*, CH43, and genetic analysis of mutacin I biosynthesis genes. *Appl Environ Microb* **66,** 3221-29 (2000).
2. Novak, J., Caufield, P.W., Miller, E.J. Isolation and Biochemical-Characterization of a Novel Lantibiotic Mutacin from *Streptococcus-Mutans*. *J Bacteriol* **176,** 4316-20 (1994).
3. Qi, F.X., Chen, P., Caufield, P.W. Purification of mutacin III from group III *Streptococcus mutans* UA787 and genetic analyses of mutacin III biosynthesis genes. *Appl Environ Microbiol* **65,** 3880-87 (1999).
4. Robson, C.L., Wescombe, P.A., Klesse, N.A., Tagg, J.R. Isolation and partial characterization of the *Streptococcus mutans* type All lantiblotic mutacin K8. *Microbiology* **153,** 1631-41 (2007).
5. Yonezawa, H., Kuramitsu, H.K. Genetic analysis of a unique bacteriocin, Smb, produced by *Streptococcus mutans* GS5. *Antimicrob Agents Chemother* **49,** 541-48 (2005).
6. Hyink, O., Balakrishnan, M., Tagg, J.R. *Streptococcus rattus* strain BHT produces both a class I two-component lantibiotic and a class II bacteriocin. *FEMS Microbiol Lett* **252,** 235-41 (2005).
7. Hillman, J.D., et al. Genetic and biochemical analysis of mutacin 1140, a lantibiotic from *Streptococcus mutans*. *Infect Immun* **66,** 2743-49 (1998).
8. MotaMeira, M., Lacroix, C., LaPointe, G., Lavoie, M.C. Purification and structure of mutacin B-Ny266: A new lantibiotic produced by *Streptococcus mutans*. *FEBS Lett* **410,** 275-79 (1997).
9. Qi, .FX., Chen, P., Caufield, P.W. The group I strain of *Streptococcus mutans*, UA140, produces both the lantibiotic mutacin I and a nonlantibiotic bacteriocin, mutacin IV. *Appl Environ Microbiol* **67,** 15-21 (2001).

10. Hale, J.D.F., Ting, Y.T., Jack, R.W., Tagg, J.R., Heng, N.C.K. Bacteriocin (mutacin) production by *Streptococcus mutans* genome sequence reference strain UA159: elucidation of the antimicrobial repertoire by genetic dissection. *Appl Environ Microbiol* **71,** 7613-17 (2005).
11. Hale, J.D., Balakrishnan, B., Tagg, J.R. Genetic basis for mutacin N and of its relationship to mutacin I. *Indian J Med Res* **119,** 247-51 (2004).
12. Xie, Z.J., Okinaga, T., Niu, G.Q., Qi, F.X., Merritt, J. Identification of a novel bacteriocin regulatory system in *Streptococcus mutans*. *Mol Microbiol* **78,** 1431-47(2010).
